# The relationship between glucose patterns in OGTT and adverse pregnancy outcomes in twin pregnancies

**DOI:** 10.1111/1753-0407.70016

**Published:** 2024-10-27

**Authors:** Wei‐Zhen Tang, Qin‐Yu Cai, Yi‐Fan Zhao, Hao‐wen Chen, Xia Lan, Xia Li, Li Wen, Ying‐Xiong Wang, Tai‐Hang Liu, Lan Wang

**Affiliations:** ^1^ Department of Obstetrics and Gynecology Women and Children's Hospital of Chongqing Medical University Chongqing China; ^2^ Department of Bioinformatics School of Basic Medical Sciences, Chongqing Medical University Chongqing China; ^3^ The Joint International Research Laboratory of Reproduction and Development Chongqing Medical University Chongqing China

**Keywords:** GDM, OGTT, pregnancy outcomes, twin pregnancy

## Abstract

**Background:**

Traditional fixed thresholds for oral glucose tolerance test (OGTT) results may inadequately prevent adverse pregnancy outcomes in twin pregnancies. This study explores latent OGTT patterns and their association with adverse outcomes.

**Methods:**

This study retrospectively analyzed 2644 twin pregnancies using latent mixture models to identify glucose level patterns (high, HG; medium, MG; and low, LG) and their relationship with maternal/neonatal characteristics, gestational age at delivery, and adverse outcomes.

**Results:**

Three distinct glucose patterns, HG, MG, and LG patterns were identified. Among the participants, 16.3% were categorized in the HG pattern. After adjustment, compared with the LG pattern, the HG pattern was associated with a 1.79‐fold, 1.66‐fold, and 1.32‐fold increased risk of stillbirth, neonatal respiratory distress, and neonatal hyperbilirubinemia, respectively. The risk of neonatal ICU admission for MG and HG patterns increased by 1.22 times and 1.32 times, respectively, compared with the LG pattern. As gestational weeks increase, although there is an overlap in the confidence intervals between the HG pattern and other patterns in the restricted cubic splines analysis, the trend suggests that pregnant women with the HG pattern are more likely to face risks of their newborns requiring neonatal intensive care unit admission, and adverse comprehensive outcomes, compared with other patterns. In addition, with age and body mass index increasing in HG mode, gestation weeks at delivery tend to be later than in other modes.

**Conclusion:**

Distinct OGTT glucose patterns in twin pregnancies correlate with different risks of adverse perinatal outcomes. The HG pattern warrants closer glucose monitoring and targeted intervention.

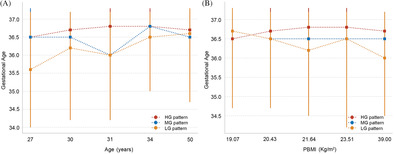

## Graphical abstract

Highlights

- Latent mixture models identified LG, MG, and HG glucose patterns in pregnant women.
- The study compared maternal and neonatal traits and adverse outcomes across patterns, analyzing outcome trends from LG to HG.
- It assessed the glucose patterns' effects on delivery timing and neonatal adverse outcomes and evaluated these impacts by maternal characteristics.

## 1 INTRODUCTION

Gestational diabetes mellitus (GDM) affects approximately 6%–10% of pregnancies and not only increases the risk of complications such as intrahepatic cholestasis of pregnancy (ICP) and infections for the expectant mother but also poses risks for the newborn, including hypoglycemia, hypocalcemia, and respiratory distress syndrome.^1^ Moreover, women with GDM have an increased likelihood of developing type 2 diabetes in the future.^2^ To reduce these complications, appropriate monitoring is essential, and the widely adopted the International Association of the Diabetes and Pregnancy Study Groups criteria (IADPSG) guidelines recommend that all pregnant women without preexisting diabetes undergo a 75 g oral glucose tolerance test (OGTT) between 24 and 28 weeks of gestation.^3^ Traditionally, pregnant women who receive normal results from an OGTT do not usually continue to monitor their blood sugar levels in the later stages of pregnancy. This practice is based on the assumption that if OGTT results are below traditional fixed thresholds, it is unlikely that the mother and fetus will suffer adverse pregnancy outcomes related to high blood sugar. However, recent findings from the Hyperglycemia and Adverse Pregnancy Outcome (HAPO) study have challenged this conventional belief. The study results clearly demonstrate that the risk of adverse pregnancy outcomes, such as cesarean delivery, neonatal hypoglycemia, and neonatal hyperinsulinemia, increases with the mother's blood sugar levels.^4^ Notably, these risks are also elevated in women whose OGTT blood sugar levels are below the diagnostic thresholds for GDM, suggesting that even lower blood sugar levels may be associated with an increased risk of adverse pregnancy outcomes.

During OGTT, identifying high‐risk individuals based solely on a single time point glucose value is challenging, especially for those who do not meet the GDM diagnostic criteria yet are still at high risk. Currently, there are no guidelines that clearly define the risk threshold for such hyperglycemia. However, different characteristics of glucose patterns derived from OGTT, such as the timing of peak and nadir, have been shown to predict the risk of developing diabetes in the general population.^5^, ^6^, ^7^, ^8^ Despite this, research on glucose patterns during OGTT in pregnancy is relatively scarce and mostly focused on singleton pregnancies.

In recent years, the incidence of twin pregnancies has increased due to the rise in maternal age at conception and the widespread use of assisted reproductive technologies.^9^ Studies suggest that the incidence of hyperglycemia and GDM is higher in twin pregnancies compared with singletons,^10^ potentially due to larger placental mass and higher levels of placental hormones,^11^, ^12^, ^13^ leading to increased insulin resistance. However, complications associated with hyperglycemia may be less clinically apparent in twin pregnancies, such as a lower incidence of macrosomia and shoulder dystocia due to slower fetal growth in the third trimester.^14^, ^15^, ^16^ Conversely, the risks of small for gestational age (SGA) infants and preterm birth are relatively higher in twin pregnancies.^17^, ^18^ At the same time, twin pregnancies may modulate the effects of hyperglycemia and GDM on the mother and fetus due to their amplified physiological changes. These changes include increased insulin resistance and a higher demand for nutrients, potentially leading to different requirements for blood glucose control. Consequently, screening and treatment standards established based on studies of singleton pregnancies may not be directly applicable to twin pregnancies.^19^, ^20^ The traditional fixed thresholds for OGTT results may no longer be sufficient to prevent adverse pregnancy outcomes in twin pregnancies. Currently, most large‐scale studies on hyperglycemia and GDM during pregnancy do not include twin pregnancies, or if they do, only a limited number of cases are considered. For instance, the landmark HAPO study did not include any twin pregnancies,^21^ limiting the generalizability of its findings. This research gap indicates a lack of comprehensive understanding of the impact and management of hyperglycemia in twin pregnancies. Against this backdrop, our study aims to fill this void by exploring the relationship between different OGTT blood glucose patterns and adverse outcomes in twin pregnancies and evaluating the potential for identifying high‐risk groups within this population. Our findings may offer new opportunities for the prevention of adverse pregnancy outcomes and provide fresh evidence for clinical practice.

## 2 MATERIALS AND METHODS

### 2.1 Ethical approval

The research received ethical clearance from the Women and Children's Hospital of Chongqing Medical University, under the approval number 2022‐011‐01. In adherence to privacy protection protocols, all data were anonymized by excluding any information that could potentially identify individual patients.

### 2.2 Data collection

All data were sourced from the electronic medical records database of the Women and Children's Hospital of Chongqing Medical University, including basic information about pregnant women and pregnancy‐related information.

### 2.3 Study design

**FIGURE 1:**
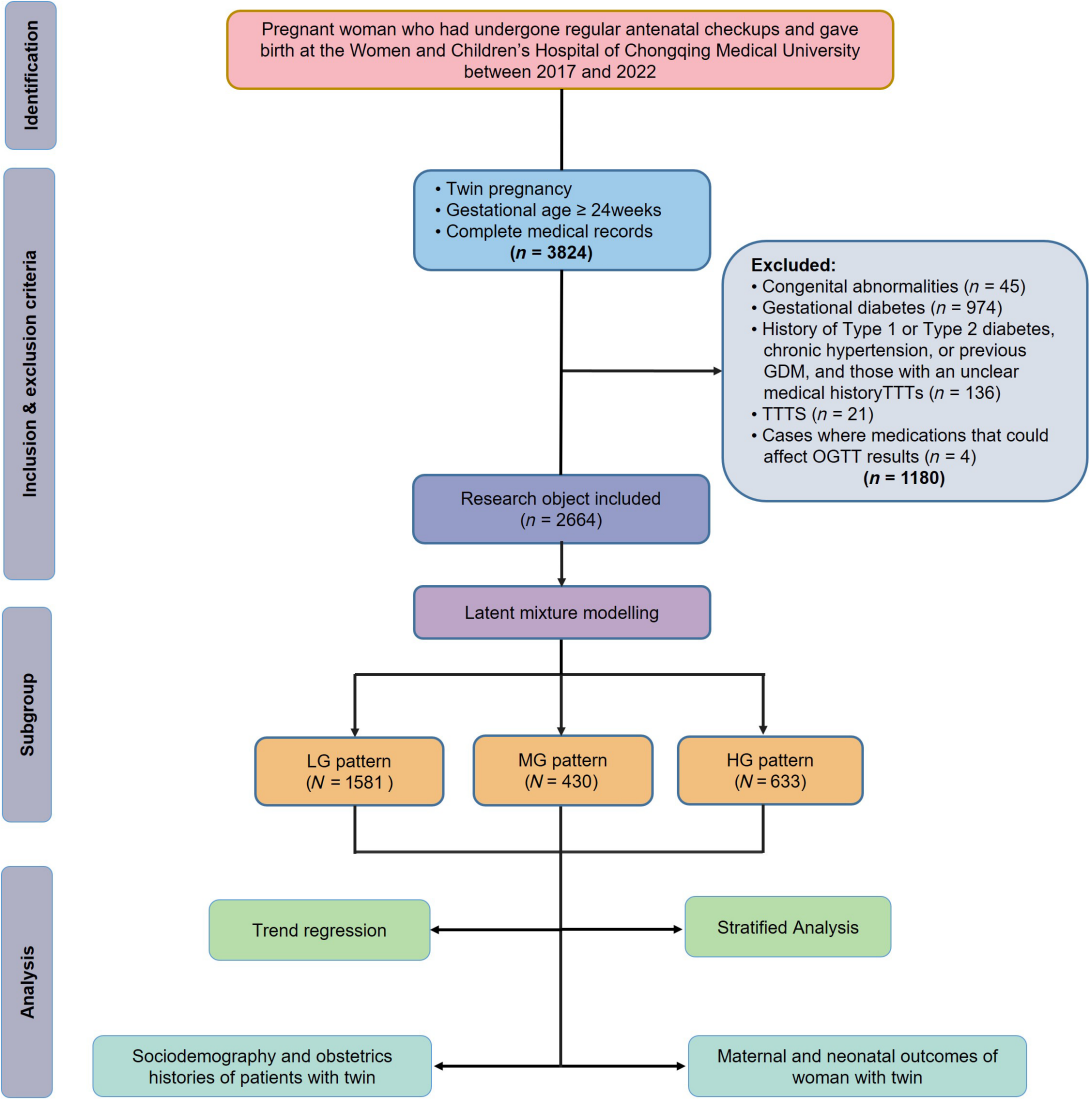
Flowchart of this retrospective cohort study.

This retrospective cohort study was conducted at the Women and Children's Hospital of Chongqing Medical University in China. The study retrospectively included pregnant women who received regular prenatal care and delivered between the years 2017 and 2022. The inclusion criteria were as follows: (i) complete medical records; (ii) twin pregnancies; and (iii) gestational age ≥24 weeks. The exclusion criteria were as follows: (i) twin pregnancies with one or both fetuses having congenital anomalies (*n* = 45); (ii) diagnosed with GDM based on the results of the OGTT during the 24–28 weeks of gestation (*n* = 974); (iii) women with a history of Type 1 or Type 2 diabetes, chronic hypertension, or previous GDM, and those with an unclear medical history (*n* = 136); (iv) cases of twin‐twin transfusion syndrome (*n* = 21); and (v) cases where medications that could affect OGTT results, such as glucocorticoids, antipsychotics, and diuretics, were used prior to screening (*n* = 4). A total of 3824 pregnant women met the inclusion criteria. After applying the exclusion criteria, 2644 cases were ultimately included in the study (Figure 1).

### 2.4 Definition

Advanced maternal age is typically defined as childbirth in women aged 35 and above.^22^, ^23^ Older women are considered to have a higher risk of GDM and are at greater risk for adverse pregnancy complications.^24^ According to Chinese guidelines, body mass index (BMI) is categorized into four classes: normal weight (BMI 18.5–23.9 kg/m^2^), overweight (BMI 24.0–27.9 kg/m^2^), and obese (BMI ≥28 kg/m^2^).^25^ In vitro fertilization (IVF) is a medical procedure whereby an egg is fertilized by sperm outside the body, typically in a laboratory dish, with the resulting embryo then transferred to the uterus for pregnancy.

### 2.5 Maternal outcomes

PROM is defined as the rupture of the amniotic sac and leakage of amniotic fluid before the onset of labor, typically diagnosed by the clinical observation of fluid passing from the cervical canal and pooling in the vagina. Preeclampsia is a pregnancy complication characterized by high blood pressure and often accompanied by proteinuria or other organ dysfunction, and its diagnosis is based on the emergence of new hypertension (blood pressure ≥ 140/90 mmHg) and/or proteinuria after 20 weeks of pregnancy. ICP is a pregnancy‐specific liver disease that is usually diagnosed based on itching (especially on the palms and soles of the feet) and elevated bile acid levels in blood tests. Postpartum hemorrhage is defined as blood loss exceeding 500 mL after vaginal delivery or 1000 mL after cesarean section.

### 2.6 Neonatal outcomes

Infants born with a birth weight below the 10th percentile for their gestational age are diagnosed as SGA. Preterm birth is defined as delivery after 28 weeks but before 37 weeks of gestation. Macrosomia is defined as a birth weight equal to or greater than 4000 g, while low birth weight is less than 2500 g. All outcome measures are based on these definitions.

### 2.7 Statistical analysis

#### 2.7.1 Latent mixture models

Latent mixture models were employed to identify subgroups sharing similar underlying glucose patterns during the OGTT at 24–28 weeks of gestation. Initially, a basic model was established, assuming that all participants shared a common glucose metabolic pattern. Subsequently, the model was expanded incrementally to consider the presence of two and three distinct latent glucose patterns. Following each model expansion, the Bayesian Information Criterion (BIC) was calculated to assess the balance between model complexity and fit. BIC is a criterion for model selection, used to compare the relative quality of different models, with a lower value indicating a preferable model. In addition to BIC, the minimum sample size for each latent class was also considered. This was to ensure that each identified subgroup had a sufficient number of samples, thereby guaranteeing the reliability and representativeness of the statistical analysis. Upon determining the optimal number of latent classes, the model was used to estimate the posterior probabilities for each participant belonging to the various glucose patterns. Participants were then assigned to the glucose pattern for which they had the highest posterior probability, indicating that they were most likely to belong to that pattern. This statistical analysis allows for changes in survey data over time and can identify heterogeneous patterns based on the three time points of the OGTT, providing a more comprehensive analysis of correlations, consistent with the OGTT pattern recognition approach in previous single pregnancy studies.^26^

#### 2.7.2 Description and statistics

Using SPSS software version 26.0 and R Studio version 4.0.2, the sociodemographic characteristics of the subjects were described. Continuous data that followed a normal distribution were expressed as mean values and standard deviations, while non‐normally distributed data were represented by median values and interquartile ranges. Further comparisons of parameters between two groups within subgroups were performed using the Mann–Whitney U test and the Chi‐square test.

By constructing a multifactorial logistic regression model, we explored the relationship between different blood glucose patterns and the risk of adverse pregnancy outcomes after adjusting for maternal age, IVF, and prepregnancy body mass index (PBMI). The trend of the risk of adverse pregnancy outcomes from the LG pattern to the HG pattern was analyzed using trend regression. Further, significant neonatal adverse outcomes from trend regression were examined using restricted cubic splines (RCS) to test for non‐linear relationships between gestational age at delivery and the incidence of these outcomes under different OGTT pattern groups, as well as to observe the trend of ORs. The reference value for gestational age at delivery was the median.

Finally, we also divided age and BMI into quintiles to explore the influence of different glucose patterns on gestational age at delivery and their trends across various age groups and BMI levels. Further stratified analyses examined the relationship between glucose patterns and adverse pregnancy outcomes under different conditions such as advanced maternal age, different BMI categories, nulliparity, whether the chorionicity was dichorionic‐diamniotic (DCDA), whether IVF treatment was received, and the presence or absence of uterine scarring. Additionally, we assessed the interaction effects between maternal characteristics and OGTT patterns on the outcomes that were significant in trend regression by including multiplicative interaction terms in the generalized linear models, and calculated the corresponding interaction *p*‐values after adjusting for the same confounding factors.

## 3 RESULTS

### 3.1 Characteristics of the three OGTT patterns

**FIGURE 2:**
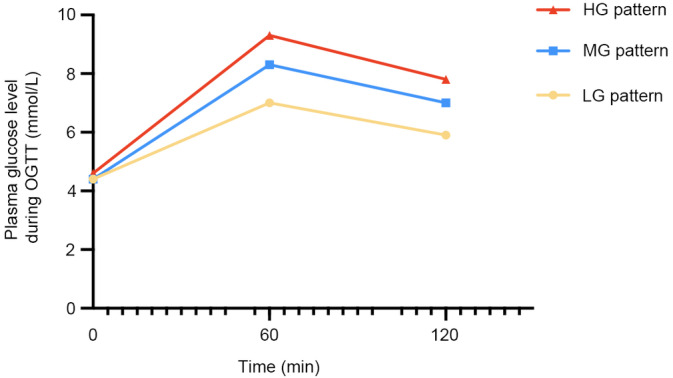
Glucose patterns during OGTT at 24‐28 weeks of gestation. The HG, MG, and LG patterns represent three distinct models of OGTT, encompassing high, medium, and low glucose levels, respectively.

Through latent class modeling, three distinct glucose patterns were identified among 2644 participants with normal OGTT results at 24–28 weeks of gestation, including high, medium, and low glucose level patterns (HG, MG, and LG patterns, respectively) (Figure 2). The HG pattern accounted for 16.3% of participants and was characterized by having the highest fasting blood glucose (FBG) levels of 4.60 (4.30–4.80) mmol/L, 1‐h glucose levels of 9.30 (9.00–9.59) mmol/L, and 2‐h glucose levels of 7.80 (7.40–8.10) mmol/L. However, a majority of participants, 59.8% (LG pattern), had lower glucose levels at these three time points with FBG at 4.40 (4.20–4.60) mmol/L, 1‐h glucose at 7.00 (6.20–7.70) mmol/L, and 2‐h glucose at 5.90 (5.30–6.40) mmol/L.

### 3.2 Maternal and neonatal characteristics and adverse pregnancy outcomes of the three OGTT patterns

In this study, the analysis revealed that the HG and MG patterns in pregnant women were associated with significantly higher maternal age and PBMI compared to the LG pattern, with p‐values less than 0.001 for both comparisons. Furthermore, the prevalence of IVF treatments among women with HG and MG patterns was significantly greater than that observed in the LG pattern group (*p* < 0.001). Among the pregnant women with the three glucose patterns, there were no significant differences in the proportions of first pregnancy, nulliparity, chorionicity, and uterine scarring. In the 24–28 weeks gestational OGTT, compared to the LG pattern, the HG pattern had significantly higher FBG, 1‐h, and 2‐h glucose levels, while only load blood glucose levels were lowest in LG group (*p* < 0.001). Additionally, there were no significant differences in the risk of adverse maternal pregnancy outcomes across the three patterns (Table 1).

**TABLE 1:** Maternal characteristics and adverse pregnancy outcomes associated with different glucose patterns during 24–28 weeks of gestation.

Variables	Overall (*N* = 2644)	LG pattern (*N* = 1581)	MG pattern (*N* = 633)	HG pattern (*N* = 430)	p‐value
Age, M (*Q*₁, *Q*₃)	31.00 (28.0, 33.0)	30.00 (28.0, 32.0)	31.00 (29.0, 34.0)	31.00 (29.0, 34.0)	<0.001*
PBMI, M (*Q*₁, *Q*₃)	21.08 (19.47, 23.03)	20.83 (19.23, 22.67)	21.34 (19.56, 23.14)	21.64 (19.95, 23.44)	<0.001*
Primigravida, *n* (%)	1300 (49.17)	788 (49.84)	309 (48.82)	203 (47.21)	0.613
Nulliparity, *n* (%)	2195 (83.02)	1314 (83.11)	533 (84.20)	348 (80.93)	0.374
IVF^a^, ^b^, *n* (%)	1847 (69.86)	1062 (67.17)	474 (74.88)	311 (72.33)	<0.001*
DCDA, *n* (%)	2174 (82.22)	1284 (81.21)	531 (83.89)	359 (83.49)	0.250
Scaruterus, *n* (%)	216 (8.17)	132 (8.35)	47 (7.42)	37 (8.60)	0.725
Thrombocytopenia, *n* (%)	124 (4.69)	82 (5.19)	28 (4.42)	14 (3.26)	0.229
Hypothyroidism, *n* (%)	243 (9.19)	147 (9.30)	55 (8.69)	41 (9.53)	0.872
Placenta accreta, *n* (%)	422 (15.96)	234 (14.80)	107 (16.90)	81 (18.84)	0.097
Placental abruption, *n* (%)	45 (1.70)	31 (1.96)	12 (1.90)	12 (1.90)	0.095
OGTT0, M (Q₁, Q₃)	4.40 (4.20, 4.60)	4.40 (4.20, 4.60)	4.40 (4.10, 4.60)	4.60 (4.30, 4.80)	<0.001*
OGTT1, M (Q₁, Q₃)	7.80 (6.80, 8.70)	7.00 (6.20, 7.70)	8.30 (7.90, 8.60)	9.30 (9.00, 9.59)	<0.001*
OGTT2, M (Q₁, Q₃)	6.50 (5.80, 7.30)	5.900 (5.30, 6.40)	7.00 (6.75, 7.50)	7.80 (7.40, 8.10)	<0.001*
TBA, M (Q₁, Q₃)	6.40 (3.70, 11.93)	6.40 (3.80, 11.70)	6.70 (3.70, 12.50)	6.45 (3.60, 12.08)	0.490
PROM, *n* (%)	531 (20.08)	308 (19.48)	133 (21.01)	90 (20.93)	0.641
PE, *n* (%)	334 (12.63)	187 (11.83)	85 (13.43)	62 (14.42)	0.282
ICP, *n* (%)	394 (14.90)	220 (13.92)	110 (17.38)	64 (14.88)	0.118
Postpartum hemorrhage, *n* (%)	119 (4.50)	75 (4.74)	21 (3.32)	23 (5.35)	0.223
MICU admission, *n* (%)	108 (4.08)	60 (3.80)	29 (4.58)	19 (4.42)	0.651
Chorioamnionitis, *n* (%)	41 (1.55)	22 (1.39)	11 (1.74)	8 (1.86)	0.713
Pelvic Inflammatory, *n* (%)	478 (18.08)	83 (19.30)	263 (16.64)	132 (20.85)	0.051

Abbreviations: DCDA, dichorionic‐diamniotic; ICP, intrahepatic cholestasis of pregnancy; IVF, in vitro fertilization; OGTT, oral glucose tolerance test; PBMI, pre‐pregnancy body mass index; PE, preeclampsia; ; PPROM, premature rupture of membranes; TBA, total bile acids; MICU admission, maternal intensive care unit admission.

* *p* < 0.05.

^a^ There was a statistically difference between LG pattern and MG pattern.

^b^ There was a statistically difference between LG pattern and HG pattern.

In terms of gestational length, the gestational weeks for the HG pattern were significantly earlier than for the LG pattern (Table 2). Regarding neonatal adverse outcomes, compared to the LG pattern, the HG pattern had a significantly higher risk of stillbirth, neonatal intensive care unit (NICU) admission, neonatal respiratory failure, and neonatal hyperbilirubinemia (*p* < 0.010; *p* = 0.003; *p* = 0.001; *p* = 0.037), and the risk was significantly higher than the MG pattern for stillbirth and neonatal respiratory failure.

**TABLE 2:** Neonatal characteristics and adverse pregnancy outcomes associated with different glucose patterns during 24–28 weeks of gestation.

Variables	Overall (*N* = 5207 )	HG pattern (*N* = 3121 )	MG pattern (*N* = 1249)	LG pattern (*N* = 837)	p‐value
Gestational week M (*Q*₁, *Q*₃)	36.70 (35.0, 37.2)	36.7 (35.3, 37.2)	36.5 (35.0, 37.2)	36.3 (34.5, 37.2)	0.010*
Birth weight, M (*Q*₁, *Q*₃)	2460.00 (2150.00, 2710.00)	2430.00 (2100.00, 2700.00)	2470.00 (2150.00, 2720.00)	2450.00 (2160.00, 2700.00)	0.123
Bodylen, M (*Q*₁, *Q*₃)	46.00 (45.00, 48.00)	46.00 (45.00, 48.00)	46.00 (45.00, 48.00)	46.00 (44.00, 48.00)	0.121
Gender *n* (%)	2697 (51.80)	1584 (50.75)	684 (54.76)	429 (51.25)	0.053
Stillbirth^b^, ^c^, *n* (%)	81 (1.56)	41 (1.31)	17 (1.36)	23 (2.75)	0.010*
Small for gestational age infant, *n* (%)	149 (2.86)	86 (2.76)	38 (3.04)	25 (2.99)	0.852
Premature delivery, *n* (%)	946 (18.17)	570 (18.26)	209 (16.73)	167 (19.95)	0.169
Low birth weight infant, *n* (%)	867 (16.65)	515 (16.50)	197 (15.77)	155 (18.52)	0.241
Polyhydramnios, *n* (%)	68 (1.31)	41 (1.31)	10 (0.80)	17 (2.03)	0.052
Oigohydramnios, *n* (%)	121 (2.32)	73 (2.34)	33 (2.64)	15 (1.79)	0.444
NICU admission^b^, *n* (%)	1532 (29.42)	865 (27.72)	389 (31.14)	278 (3.32)	0.003*
Neonatal respiratory failure^b^, ^c^, *n* (%)	323 (6.20)	179 (5.74)	69 (5.52)	75 (8.96)	0.001*
Neonatal respiratory distress, *n* (%)	291 (5.59)	164 (5.25)	69 (5.52)	58 (6.93)	0.172
Neonatal pneumonia, *n* (%)	257 (4.94)	145 (4.65)	60 (4.80)	52 (6.21)	0.173
Neonatal enteritis, *n* (%)	58 (1.11)	38 (1.22)	15 (1.20)	5 (0.60)	0.317
Severe infections in newborns, *n* (%)	77 (1.48)	41 (1.31)	24 (1.92)	12 (1.43)	0.311
Neonatal purpura, *n* (%)	44 (0.85)	28 (0.90)	8 (0.64)	8 (0.96)	0.640
Neonatal hypoglycemia, *n* (%)	159 (3.05)	101 (3.24)	30 (2.40)	28 (3.35)	0.303
Neonatal hyperbilirubinemia^b^, *n* (%)	678 (13.02)	381 (12.21)	167 (13.37)	130 (15.53)	0.037*
Neonatal hypoproteinemia, *n* (%)	72 (1.38)	49 (1.57)	13 (1.04)	10 (1.19)	0.368
Neonatal hyperlactacidemia, *n* (%)	122 (2.34)	75 (2.40)	28 (2.24)	19 (2.27)	0.949

Abbreviation: NICU admission, neonatal intensive care unit admission.

* *p* < 0.05.

^b^ There was a statistically difference between LG pattern and HG pattern.

^c^ There was a statistically difference between MG pattern and HG pattern.

After adjusting for potential confounders, we found that the risk of ICP in the MG pattern was 1.37 times that of the LG pattern, while there was no significant difference in risk between the HG pattern and the LG pattern. In terms of neonatal outcomes, compared to the LG pattern, the risks of stillbirth, neonatal respiratory failure, and neonatal hyperbilirubinemia in the HG pattern increased by 1.79 times, 1.66 times, and 1.32 times, respectively. For the risk of NICU admission, the MG and HG patterns had an increased risk of 1.22 times and 1.32 times, respectively, compared to the LG pattern (Table 3). In trend regression analyses, the risks of stillbirth, NICU admission, neonatal respiratory failure, and neonatal hyperbilirubinemia were all statistically significant, and after adjusting for potential confounders, the p for trend remained significant for NICU admission (adjusted odds ratio [aOR]: 1.16 [1.07–1.26]); Neonatal respiratory failure (aOR: 1.25 [1.09–1.45]); neonatal hyperbilirubinemia (aOR: 1.15 [1.03–1.27]).

**TABLE 3:** Logistic regression and trend analysis exploring adverse pregnancy outcomes associated with OGTT glucose patterns at 24–28 weeks of gestation.

Outcomes	Crude OR (95% CI)	p‐value	Adjusted OR (95% CI)	p‐value
PROM				
LG pattern	Reference		Reference	
MG pattern	1.10 (0.88–1.38)	0.416	1.14 (0.90–1.43)	0.278
HG pattern	1.09 (0.84–1.42)	0.504	1.15 (0.88–1.51)	0.299
P for trend	1.06(0.93–1.20)	0.398	1.08 (0.95–1.23)	0.214
PE				
LG pattern	Reference		Reference	
MG pattern	1.16 (0.88–1.52)	0.300	1.08 (0.82–1.43)	0.569
HG pattern	1.26 (0.92–1.71)	0.149	1.15 (0.84–1.58)	0.373
P for trend	1.13 (0.97–1.31)	0.114	1.08 (0.93–1.25)	0.344
ICP				
LG pattern	Reference		Reference	
MG pattern	1.30 (1.01–1.67)	0.039*	1.37 (1.06–1.76)	0.016*
HG pattern	1.08 (0.80–1.46)	0.609	1.18 (0.87–1.60)	0.298
P for trend	1.08 (0.94–1.24)	0.262	1.13 (0.98–1.30)	0.094
Postpartum hemorrhage				
LG pattern	Reference		Reference	
MG pattern	0.69 (0.42–1.13)	0.139	0.63 (0.38–1.04)	0.070
HG pattern	1.13 (0.70–1.83)	0.606	1.03 (0.63–1.68)	0.894
P for trend	1.00 (0.78–1.27)	0.981	0.95 (0.74–1.22)	0.666
MICU admission				
LG pattern	Reference		Reference	
MG pattern	1.22 (0.77–1.91)	0.395	1.15 (0.73–1.83)	0.540
HG pattern	1.17 (0.69–1.99)	0.555	1.08 (0.63–1.85)	0.778
P for trend	1.10 (0.86–1.41)	0.435	1.06 (0.82–1.36)	0.668
Chorioamnionitis				
LG pattern	Reference		Reference	
MG pattern	1.25 (0.60–2.60)	0.544	1.25 (0.60–2.62)	0.553
HG pattern	1.34 (0.59–3.04)	0.478	1.37 (0.60–3.14)	0.455
P for trend	1.17 (0.79–1.73)	0.424	1.18 (0.79–1.76)	0.410
Stillbirth				
LG pattern	Reference		Reference	
MG pattern	1.04 (0.59–1.83)	0.903	0.90 (0.50–1.60)	0.709
HG pattern	2.09 (1.25–3.51)	0.005*	1.79 (1.05–3.03)	0.032*
P for trend	1.41 (1.08–1.84)	0.011*	1.30 (0.99~ 1.71)	0.063
NICU admission				
LG pattern	Reference		Reference	
MG pattern	1.18 (1.02–1.36)	0.025*	1.22 (1.05–1.41)	0.007*
HG pattern	1.27 (1.08–1.49)	0.004*	1.32 (1.12–1.56)	<0.001*
P for trend	1.14 (1.05–1.23)	0.001*	1.16 (1.07–1.26)	<0.001*
Small for gestational age infant				
LG pattern	Reference		Reference	
MG pattern	1.11 (0.75–1.63)	0.608	1.13 (0.76–1.67)	0.551
HG pattern	1.07 (0.68–1.68)	0.766	1.06 (0.67–1.67)	0.815
P for trend	1.05 (0.85–1.30)	0.671	1.04 (0.84–1.30)	0.697
Premature delivery				
LG pattern	Reference		Reference	
MG pattern	0.90 (0.76–1.07)	0.231	0.92 (0.77–1.10)	0.367
HG pattern	1.10 (0.90–1.33)	0.350	1.12 (0.92–1.36)	0.262
P for trend	1.02 (0.93–1.12)	0.675	1.03 (0.94–1.14)	0.490
Low birth weight infant				
LG pattern	Reference		Reference	
MG pattern	0.95 (0.79–1.13)	0.552	0.98 (0.82–1.18)	0.841
HG pattern	1.13 (0.93–1.38)	0.226	1.17 (0.96–1.43)	0.123
P for trend	1.04 (0.95–1.15)	0.392	1.07 (0.97–1.17)	0.202
Neonatal respiratory failure				
LG pattern	Reference		Reference	
MG pattern	0.96 (0.72–1.28)	0.783	1.00 (0.75–1.33)	0.990
HG pattern	1.59 (1.20–2.11)	0.001*	1.66 (1.25–2.22)	<0.001*
P for trend	1.22 (1.06–1.41)	0.006*	1.25 (1.09–1.45)	0.002*
Neonatal respiratory distress				
LG pattern	Reference		Reference	
MG pattern	1.05 (0.79–1.41)	0.723	1.08 (0.80–1.44)	0.617
HG pattern	1.32 (0.97–1.80)	0.077	1.36 (0.99–1.86)	0.058
P for trend	1.14 (0.98–1.32)	0.099	1.15 (0.99~ 1.34)	0.072
Neonatal pneumonia				
LG pattern	Reference		Reference	
MG pattern	1.04 (0.76–1.41)	0.826	1.03 (0.76–1.41)	0.837
HG pattern	1.34 (0.97–1.86)	0.079	1.33 (0.95–1.85)	0.093
P for trend	1.14 (0.97–1.34)	0.111	1.14 (0.97–1.34)	0.127
Neonatal enteritis				
LG pattern	Reference		Reference	
MG pattern	0.99 (0.54–1.80)	0.963	1.01 (0.55–1.85)	0.980
HG pattern	0.48 (0.19–1.22)	0.125	0.50 (0.19–1.28)	0.146
P for trend	0.77 (0.53–1.13)	0.178	0.78 (0.53–1.15)	0.214
Severe infections in newborns				
LG pattern	Reference		Reference	
MG pattern	1.08 (0.56–2.06)	0.822	1.49 (0.89–2.50)	0.127
HG pattern	1.47 (0.89–2.44)	0.137	1.12 (0.58–2.16)	0.730
P for trend	1.11 (0.83–1.48)	0.493	1.13 (0.84–1.51)	0.430
Neonatal purpura				
LG pattern	Reference		Reference	
MG pattern	0.71 (0.32–1.57)	0.398	0.75 (0.34–1.66)	0.471
HG pattern	1.05 (0.48–2.31)	0.902	1.12 (0.50–2.50)	0.778
P for trend	0.97 (0.65–1.44)	0.865	1.00 (0.67–1.50)	0.987
Neonatal hypoglycemia				
LG pattern				
MG pattern	0.74 (0.49–1.11)	0.145	0.72 (0.47–1.09)	0.124
HG pattern	1.02 (0.67–1.56)	0.928	0.97 (0.63–1.49)	0.876
P for trend	0.96 (0.78–1.18)	0.687	0.93 (0.75–1.16)	0.538
Neonatal hyperbilirubinemia				
LG pattern	Reference		Reference	
MG pattern	1.11 (0.91–1.35)	0.297	1.13 (0.93–1.38)	0.216
HG pattern	1.30 (1.05–1.61)	0.017*	1.32 (1.06–1.64)	0.013*
P for trend	1.14 (1.02–1.26)	0.016*	1.15 (1.03–1.27)	0.012*
Neonatal hypoproteinemia				
LG pattern	Reference		Reference	
MG pattern	0.66 (0.36–1.22)	0.184	0.66 (0.36–1.23	0.195
HG pattern	0.75 (0.38–1.48)	0.404	0.72 (0.36–1.45	0.363
P for trend	0.82 (0.59–1.14)	0.231	0.81 (0.58–1.13)	0.217
Neonatal hyperlactacidemia				
LG pattern	Reference		Reference	
MG pattern	0.93 (0.60–1.44)	0.749	0.90 (0.58–1.40)	0.643
HG pattern	0.93 (0.56–1.55)	0.780	0.87 (0.52–1.45)	0.588
P for trend	0.96 (0.75–1.22)	0.726	0.93 (0.72–1.18)	0.536

Abbreviations: ICP, intrahepatic cholestasis of pregnancy; MICU admission, maternal intensive care unit admission; PE, preeclampsia; PPROM, premature rupture of membranes.

^a^ OR values were adjusted for maternal age, IVF and pre‐pregnancy BMI.

* *p* < 0.05.

### 3.3 The association between gestational age at delivery and adverse pregnancy outcomes under different OGTT patterns in RCS nested logistic regression

In the analysis using RCS across different OGTT patterns, the gestational age at delivery significantly affected the risk of NICU admission, neonatal respiratory failure, neonatal hyperbilirubinemia, and composite adverse outcomes (*p* < 0.001) for all three patterns. There was a significant non‐linear relationship between gestational age at delivery and each adverse neonatal outcome across the three different OGTT patterns (*p* for nonlinearity <0.001). Specifically, regardless of gestational week, infants from the HG pattern group were more likely to require admission to the NICU and to experience respiratory distress compared to those from the LG and MG pattern groups. Additionally, the MG pattern group exhibited a higher trend in the risk of jaundice across all gestational weeks when compared with the LG and HG patterns. Although the overlapping confidence intervals preclude statistical certainty, these findings suggest a potential association that warrants further investigation. The LG pattern group showed the lowest trend in both NICU admission rates and the risk of hyperbilirubinemia. Moreover, in terms of respiratory failure, the MG pattern group presented a lower risk trend compared with the LG and HG pattern groups. However, in our stratified analysis based on OGTT patterns, we encountered a problem of insufficient sample size, particularly with cases involving neonatal respiratory failure and hyperbilirubinemia. This limitation weakened our ability to explore the relationship between gestational age at delivery and adverse neonatal outcomes across different OGTT patterns. Furthermore, the scarcity of cases involving deliveries before 34 weeks posed a challenge for modeling the risk of adverse neonatal outcomes in early gestation using RCS. The paucity of data led to a broad range of estimates for the OR, potentially diminishing the precision and reliability of these estimates (Figure S1).

### 3.4 Risk of adverse maternal and neonatal pregnancy outcomes across three OGTT patterns under diverse maternal characteristics

**FIGURE 3:**
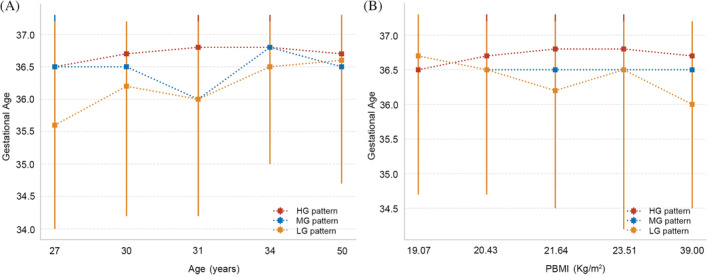
(A) The impact of glucose patterns on gestational weeks at delivery for pregnant women of different age groups. (B) The impact of glucose patterns on gestational weeks at delivery for pregnant women with different BMI levels.

Observations indicated that as the glucose pattern in the OGTT shifted from an LG pattern to an HG pattern, there was a corresponding increase in the gestational weeks at the time of delivery. To delve deeper into the effects of OGTT patterns on the gestational weeks at delivery for women across different age and BMI stratifications, this study categorized both age and BMI into five distinct quintiles for assessment. In the analysis stratified by age, regardless of the age group, women in the HG pattern group exhibited the longest gestational weeks at delivery (Figure 3). In the LG pattern group, women below the age of 34 had the shortest gestational weeks at delivery; however, once surpassing the age of 34, their gestational weeks exceeded those of women in the MG pattern group. In the analysis stratified by BMI, it was observed that within the LG pattern group, the higher the BMI of the women, the earlier the gestational weeks at delivery. In contrast, women in the HG pattern group maintained consistently higher gestational weeks at delivery, irrespective of their BMI levels (Tables S1 and S2).

Further stratified analyses were conducted for adverse pregnancy outcomes with significant trend regression. Regarding NICU admission, the OGTT patterns had a significant impact on women of normal weight and those who were obese. In terms of neonatal respiratory failure, the OGTT patterns significantly affected overweight, obese women, non‐nulliparous women, primigravid women, and those who had not undergone IVF treatments. For neonatal hyperbilirubinemia, the OGTT patterns had a significant impact on women of normal weight, primigravid women, and those who had not undergone IVF treatments. In addition, it was also discovered that in non‐elderly women, those with DCDA twin pregnancies, and those without uterine scarring, the OGTT patterns had a significant impact on the risk of the aforementioned three neonatal diseases. Finally, the study conducted multiplicative interaction analyses to examine the interplay between OGTT patterns and various maternal characteristics. The findings revealed significant interactions: for all three neonatal outcomes, there was a notable interaction between OGTT patterns and PBMI (*p* for interaction <0.05). Furthermore, significant interactions were observed between OGTT patterns and primigravid status in relation to NICU admissions and neonatal hyperbilirubinemia. Additionally, significant interactions were found between OGTT patterns and nulliparity status for the risks of neonatal respiratory failure and neonatal hyperbilirubinemia. On the other hand, the presence of scaruterus interacted significantly with OGTT patterns in the context of NICU admissions (*p* for interaction = 0.041). These results underscore the critical role of maternal characteristics in modulating the sensitivity of neonatal outcomes to OGTT patterns, with variations observed across different maternal features and neonatal outcomes (Table 4).

**TABLE 4:** Stratified analysis of adverse pregnancy outcomes associated with OGTT glucose patterns at 24–28 weeks of gestation.

		NICU admission					Neonatal respiratory failure					Neonatal hyperbilirubinemia				
	N	OR (95%CI)	P‐value	^a^OR (95%CI)	P‐value	P‐interaction	OR (95%CI)	P‐value	aOR(95%CI)	P‐value	P‐interaction	OR (95%CI)	P‐value	^a^OR (95%CI)	P‐value	P‐interaction
Age						0.586					0.472					0.170
≤35	4695	1.19 (1.09–1.29)	0.001*	1.190 (1.10, 1.29)	0.001*		1.27 (1.09–1.47)	0.002*	1.25 (1.08, 1.46)	0.003*		1.16 (1.04–1.29)	0.011*	1.16 (1.03,1.29)	0.011*	
>35	512	0.85 (0.66–1.08)	0.170	0.84 (0.66, 1.06)	0.145		0.85 (0.51–1.41)	0.523	0.79 (0.46, 1.33)	0.368		0.92 (0.68–1.25)	0.607	0.01 (0.67,1.23)	0.535	
PBMI						0.018*					0.001*					0.013*
<18.5 kg/m^2^	697	1.05 (0.83–1.34)	0.667	1.22 (0.95, 1.57)	0.122		1.07 (0.67–1.71)	0.768	1.18 (0.73, 1.92)	0.496		0.85 (0.60–1.20)	0.362	0.97 (0.68, 1.39)	0.857	
18.5–23.9 kg/m^2^	3672	1.14 (1.04–1.25)	0.005*	1.15 (1.05, 1.27)	0.003*		1.11 (0.93–1.32)	0.269	1.13 (0.95, 1.36)	0.177		1.20 (1.06–1.36)	0.003*	1.21 (1.07, 1.37)	0.003*	
24.0–27.9 kg/m^2^	746	1.11 (0.91–1.36)	0.294	1.17 (0.96, 1.44)	0.124		1.35 (0.96–1.89)	0.085	1.54 (1.09, 2.18)	0.015*		0.96 (0.74–1.26)	0.790	1.01 (0.77, 1.33)	0.928	
≥28 kg/m^2^	92	1.91 (1.12–3.27)	0.018*	1.93 (1.09,3.40)	0.023*		4.13(1.61–10.61)	0.003*	3.99 (1.53,10.39)	0.005*		1.33 (0.72–2.45)	0.366	1.26 (0.65,2.44)	0.495	
Primigravida						0.039*					0.498					0.045*
No	2644	1.19 (1.07–1.33)	0.001*	1.15 (1.02,1.29)	0.018*		1.20 (0.98–1.47)	0.072	1.28 (1.04,1.58)	0.019*		1.19 (1.03–1.38)	0.019*	1.11 (0.95,1.29)	0.184	
Yes	2563	1.10 (0.98–1.23)	0.099	1.20 (1.07,1.34)	0.002*		1.23 (1.00–1.50)	0.049*	1.21 (0.99, 1.49)	0.066		1.08 (0.93–1.26)	0.305	1.18 (1.02,1.37)	0.028*	
Nulliparity						0.328					0.025*					0.006*
No	877	1.50 (1.25–1.80)	<0.001*	1.48 (1.23,1.78)	<0.001*		1.33 (0.94–1.88)	0.109	1.23 (0.86,1.77)	0.257		1.23 (0.96–1.56)	0.101	1.12 (0.87,1.43)	0.397	
Yes	4330	1.08 (0.99–1.18)	0.090	1.11 (1.02, 1.22)	0.017*		1.19 (1.02–1.40)	0.027*	1.25 (1.07, 1.47)	0.006*		1.12 (0.99–1.25)	0.064	1.16 (1.03, 1.30)	0.016*	
DCDA						0.849					0.864					0.308
No	466	1.12 (0.87–1.45)	0.369	1.19 (0.91, 1.56)	0.204		1.06 (0.70–1.62)	0.780	1.11 (0.71, 1.3)	0.651		1.02 (0.74–1.41)	0.917	1.03 (0.73, 1.44)	0.874	
Yes	2157	1.16 (1.03–1.31)	0.018*	1.17 (1.03, 1.32)	0.014*		1.36 (1.08–1.72)	0.010*	1.37 (1.08, 1.74)	0.009*		1.27 (1.08–1.49)	0.003*	0.87 (1.09, 1.51)	0.003*	
IVF						0.125					0.158					0.410
No	1572	1.15 (1.00–1.33)	0.053	1.18 (1.02, 1.36)	0.031*		1.33 (1.04–1.71)	0.025*	1.29 (1.00, 1.66)	0.048*		1.20 (1.00–1.44)	0.046*	1.23 (1.02, 1.48)	0.031*	
Yes	3635	1.15 (1.04–1.26)	0.005*	1.17 (1.06,1.28)	0.002*		1.17 (0.98–1.39)	0.079	1.17 (0.98, 1.40)	0.077		1.12 (0.98–1.27)	0.092	1.11 (0.97, 1.26)	0.129	
Scaruterus						0.041*					0.470					0.219
No	2409	1.13 (1.01–1.27)	0.041*	1.16 (1.03, 1.30)	0.015*		1.32 (1.07–1.62)	0.010*	1.35 (1.09, 1.68)	0.006*		1.23 (1.06–1.43)	0.006*	1.27 (1.10, 1.48)	0.002*	
Yes	214	1.36 (0.94–1.96)	0.100	1.37 (0.95, 1.99)	0.096		0.76 (0.32–1.76)	0.518	0.75 (0.32, 1.73)	0.497		0.93 (0.53–1.63)	0.802	0.90 (0.510, 1.58)	0.706	

Abbreviations: PBMI, pre‐pregnancy body mass index; IVF, in vitro fertilization; DCDA, dichorionic‐diamniotic.

^a^ OR values were adjusted for maternal age, IVF and pre‐pregnancy BMI.

* *p* < 0.05.

## 4 DISCUSSION

In this study, three distinct glucose patterns (high, medium, and low glucose levels: HG, MG, and LG patterns) were identified among normal OGTT values between 24 and 28 weeks of gestation using latent class models, revealing significant differences in demographic and obstetric characteristics among these patterns (*p* < 0.001). We observed that 23.9% of the participants exhibited the HG pattern, which was associated with increased risks of adverse neonatal outcomes such as stillbirth, neonatal respiratory failure, NICU admission, and neonatal hyperbilirubinemia, with no significant differences in adverse maternal outcomes. Notably, compared with the LG pattern, the MG pattern showed a significantly increased risk of ICP, an observation not seen in the HG pattern, suggesting a need for further research to elucidate the underlying mechanisms. It was also found that as the pattern shifted from LG to HG, there was a corresponding delay in the number of gestation weeks at delivery (*p* < 0.001), which may partly explain the increased risk of adverse neonatal outcomes in the HG pattern. Additionally, we noted a trend of earlier gestational weeks at delivery with increasing BMI within the HG pattern, indicating that overweight or obese pregnant women might be at a higher risk of preterm delivery under the HG pattern. We observed that compared to the LG model, the gestational weeks for the MG and HG models were extended by 0.2 and 0.4 weeks, respectively. These findings align with those from studies on glucose models in singleton pregnancies,^26^ where MG and HG models were associated with a higher risk of large for gestational age infants. The same studies also indicated that MG and HG models correlate with an increased risk of macrosomia. However, our study did not show a significant difference in neonatal weight across different glucose models. This phenomenon may be related to the unique aspects of twin pregnancies. Compared with singletons, twins generally grow at a slower rate. Therefore, the association between high blood glucose and pregnancy complications such as macrosomia may not be as pronounced in twin pregnancies. As pointed out in the research by Hiersch et al.,^27^ the unique growth pattern of twins may attenuate the link between high blood glucose and macrosomia. Additionally, the perspective provided by Alexandra that high blood glucose might be beneficial for the weight outcomes of twin pregnancies^28^ offers a new angle on this relationship. In summary, our findings provide insights into the pattern of glucose metabolism in twin pregnancies. The study also indicates that under the HG pattern, the risk of adverse neonatal outcomes is higher across all gestational weeks, and the impact of gestational weeks on outcomes exhibits a non‐linear characteristic. This provides important clinical guidance in managing pregnant patients with diabetes, especially in determining the optimal timing of delivery to minimize the risk of adverse neonatal outcomes. Our study also assessed the impact of the three OGTT patterns on adverse maternal and neonatal pregnancy outcomes under different maternal characteristics through stratified analysis. We found that the OGTT patterns had significant effects on adverse pregnancy outcomes in women who were not of advanced age, had DCDA chorionicity, and had no uterine scarring, indicating the broad applicability of OGTT patterns in identifying high‐risk groups. Furthermore, our findings suggest that obesity status, nulliparity, primigravidity, and IVF treatment could be key factors affecting the identification of high‐risk groups based on OGTT patterns.

OGTT serves as a useful tool for assessing glucose regulation in the body. The current focus of gestational OGTT is on individual glucose values at each time point, and previous studies have reported associations between abnormal OGTT values and adverse pregnancy outcomes and postpartum complications.^29^, ^30^, ^31^ However, identifying high‐risk individuals based solely on single time‐point glucose values has its limitations. Previous research has suggested that the heterogeneity of OGTT glucose patterns in the general population may reflect the risk of future diabetes.^5^, ^6^, ^7^ Correspondingly, our study focuses on glucose patterns of normal OGTT values during twin pregnancies, revealing that even within normal OGTT values at 24–28 weeks gestation, there exist three distinct glucose patterns. We also found that the HG pattern is characterized by relatively higher FBG and 1‐ and 2‐h glucose levels within the normal OGTT, where FBG can reflect glucose metabolism under baseline conditions, typically associated with steady‐state β‐cell dysfunction and chronic low β‐cell mass, while 1‐ and 2‐h glucose levels can measure post‐glucose load metabolism and are more likely associated with environmental factors, including diet and physical exercise.^32^, ^33^, ^34^ In our study, significant differences were observed in all FBG and 1‐ and 2‐h glucose levels between the three patterns, implying that steady‐state β‐cell function, baseline insulin secretion, and peripheral insulin sensitivity may differ among the patterns.

Although current IADPSG diagnostic criteria and guidelines recommend special attention to diet, exercise, and even insulin intervention when any OGTT marker exceeds the threshold,^3^ our findings suggest that even a hyperglycemic pattern that does not meet the GDM diagnostic criteria still poses risks to twin pregnancies, particularly for neonatal outcomes. The current IADPSG standards do not differentiate between singleton and twin pregnancies in screening and diagnosing GDM, and the HAPO study, on which these standards are based, did not include any twin pregnancies. Given that the physiological effects of singleton and twin pregnancies may significantly differ,^19^, ^20^ future research should explore new methods for screening and treatment of GDM in twin pregnancies. At the same time, considering that under current standards, pregnant women with normal OGTT results do not continue to monitor their blood glucose levels in the late stages of pregnancy, we propose that for those with higher blood glucose levels at all time points but with normal OGTT results—specifically, the HG and MG patterns—it would be prudent to continue monitoring blood glucose levels after the 24th–28th week of pregnancy. This continuous monitoring should be coupled with timely dietary adjustments and increased physical activity to reduce the risk of adverse pregnancy outcomes. Our research indicates that there is still a risk associated with elevated blood glucose levels that do not meet the current diagnostic thresholds for GDM, even when the OGTT results are within the normal range. This risk is particularly pronounced in twin pregnancies due to the higher metabolic demands and the narrower margin for error in glucose management.

### 4.1 Limitations

Despite these findings, the study is not without its limitations. First, it is retrospective in design, which may be subject to limitations in data collection. Obtaining and quantifying data on diet, exercise, and psychological status in women with twin pregnancies is challenging, limiting our ability to fully explore confounding effects of these variables. In addition, the absence of records of gastrointestinal surgery may affect glucose absorption and alter OGTT results, which is a limitation. However, it is important to note that most gastrointestinal surgeries that have a significant impact on glucose metabolism, such as bariatric surgery, are not usually performed during pregnancy due to their inherent risks, suggesting that confusion from this source may be minor. Secondly, there was no long‐term postpartum follow‐up for all women and newborns across different OGTT patterns, such as future maternal cardiovascular diseases, diabetes, and the impact on neurodevelopment in infants.^2^, ^35^ Finally, although latent mixture models have advantages in revealing the heterogeneity and complexity of glucose metabolism during pregnancy and can provide valuable predictive insights, their use is not unlimited. In particular, the complexity of latent mixture models has the potential to lead to overfitting, especially when there are many parameters, and also presents computational challenges. In addition, while BIC helps to avoid overfitting through parametric penalties, its sensitivity to sample size and progressive approximation in small sample situations may limit its effectiveness in model selection. Therefore, when using latent mixture models to study glucose metabolism patterns during pregnancy, BIC must be carefully used to guide model selection to ensure that a model that accurately reflects the data structure is not overly complex. At the same time, the paucity of data on extremely preterm births, coupled with insufficient records of neonatal morbidity, constrains the initial phase of the RCS analysis. To elucidate the relationship between different OGTT gestational patterns and adverse neonatal outcomes, future studies will require multicentric data that can provide a more robust dataset. This will enable a more comprehensive understanding of the potential implications and guide clinical decision‐making with greater precision.

## 5 CONCLUSION

In summary, our study has revealed a potential link between the heterogeneity of glucose patterns during the OGTT at 24–28 weeks of gestation and adverse pregnancy outcomes. Specifically, we have observed that different glucose patterns during the OGTT are associated with adverse neonatal outcomes such as NICU admissions, neonatal respiratory failure, and neonatal hyperbilirubinemia. It should also be considered that pregnant women with relatively high glucose levels at all points of a normal OGTT may be at risk and should have their glucose levels closely monitored after the OGTT. Further multicenter prospective studies are needed to confirm and validate our findings.

## AUTHOR CONTRIBUTIONS

Conceptualization, Wei‐Zhen Tang, Qin‐Yu Cai, Yi‐Fan Zhao, Hao‐wen Chen, Tai‐Hang Liu, and Lan Wang; methodology, Wei‐Zhen Tang, Xia Li, Tai‐Hang Liu, and Lan Wang; software, Wei‐Zhen Tang, Xia Lan, and Lan Wang; validation, Qin‐Yu Cai, Li Wen, Ying‐Xiong Wang, and Lan Wang; investigation, Wei‐Zhen Tang, Li Wen, and Lan Wang; data curation, Wei‐Zhen Tang, Yi‐Fan Zhao, Xia Li, Ying‐Xiong Wang, Tai‐Hang Liu, and Lan Wang; original draft preparation, Wei‐Zhen Tang, Qin‐Yu Cai, and Tai‐Hang Liu; writing‐review and editing, Xia Lan, Xia Li, Tai‐Hang Liu, and Lan Wang; visualization, Wei‐Zhen Tang, Qin‐Yu Cai, Hao‐wen Chen, and Li Wen; supervision, Hao‐wen Chen, Yi‐Fan Zhao, Tai‐Hang Liu, and Lan Wang; project administration, Wei‐Zhen Tang, Ying‐Xiong Wang, Tai‐Hang Liu, and Lan Wang; funding acquisition Lan Wang. All authors have read and agreed to the published version of the manuscript.

## FUNDING INFORMATION

This work was funded by the Natural Science Foundation of Chongqing (No. CSTB2023NSCQ‐MSX0384) and the Yingyao Program of Chongqing Medical University.

## CONFLICT OF INTEREST STATEMENT

The authors declare no conflicts of interest.

## Supporting information


**Table S1.** The relationship between glucose patterns and gestational weeks at delivery under different maternal characteristics.
**Table S2.** The Relationship between glucose patterns and gestational weeks at delivery under different maternal characteristics.
**Figure S1.** The association between the gestational week of delivery in twin pregnancies under different blood glucose patterns and the ORs for various neonatal outcomes was determined by restricted cubic spline fitting. (A) For NICU admission. (B) For neonatal respiratory failure. (C) For neonatal hyperbilirubinemia. The OR reference level was the median week of gestation at delivery. The reference line is based on the median gestational week, and y‐axis is set to 1. These curves have been adjusted for maternal age, PBMI, nulliparity, and primigravida status.

## Data Availability

The data underlying this article will be provided by the corresponding author upon reasonable request.
