# Supplementary material for: The relationship between glucose patterns in OGTT and adverse pregnancy outcomes in twin pregnancies
Source: J Diabetes. 2024 Oct 27;16(10):e70016. doi: 10.1111/1753-0407.70016 (PMC11513445; doi:10.1111/1753-0407.70016)
Supplement: Supplementary file 1 — Table S1. The relationship between glucose patterns and gestational weeks at delivery under different maternal characteristics. Table S2. The Relationship between glucose patterns and gestational weeks at delivery under different maternal characteristics. Figure S1. The association between the gestational week of delivery in twin pregnancies under different blood glucose patterns and the ORs for various neonatal outcomes was determined by restricted cubic spline fitting. (A) For NICU admission. (B) For neonatal respiratory failure. (C) For neonatal hyperbilirubinemia. The OR reference level was the median week of gestation at delivery. The reference line is based on the median gestational week, and y‐axis is set to 1. These curves have been adjusted for maternal age, PBMI, nulliparity, and primigravida status. [file JDB-16-e70016-s001.docx]

**The relationship between glucose patterns in OGTT and adverse pregnancy outcomes in twin pregnancies**

Wei-Zhen Tang ^a, b,¶^, Qin-Yu Cai ^a, b,¶^, Yi-Fan Zhao ^b, c^, Hao-wen Chen ^a^, Xia Lan ^a^, Xia Li ^b, c^, Li Wen ^a^, Yao Zhang^a^, Ying-Xiong Wang ^b, c,^*, Tai-Hang Liu ^b, c,^*, Lan Wang ^a,^*

**Affiliations:**

^a^ Department of Obstetrics and Gynecology, Women and Children**'**s Hospital of Chongqing Medical University, Chongqing 401147, China.

^b^ Department of Bioinformatics, School of Basic Medical Sciences, Chongqing Medical University, Chongqing 400016, China.

^c^ The Joint International Research Laboratory of Reproduction and Development, Chongqing Medical University, Chongqing, 400016, China.

***Correspondence:**

* Ying-Xiong Wang (yxwang@cqmu.edu.cn); Tai-Hang Liu (liuth@cqmu.edu.cn); Lan Wang (xwanglan@163.com)

Box 197, Chongqing Medical University, No.1 Yixueyuan Rd, Chongqing, 400016, PR China. Tel.: +86 023 68485868.

**Supplementary Table 1.** The relationship between glucose patterns and gestational weeks at delivery under different maternal characteristics.

| **Age** | **Total(N)** | **Total (Median(Q1~Q3))** | **LG pattern**  **(Median(Q1~Q3))** | **MG pattern**  **(Median(Q1~Q3))** | **HG pattern**  **(Median(Q1~Q3))** | **PBMI** | **Total(N)** | **Total (Median(Q1~Q3))** | **LG pattern (Median(Q1~Q3))** | **MG pattern (Median(Q1~Q3))** | **HG pattern (Median(Q1~Q3))** |
| --- | --- | --- | --- | --- | --- | --- | --- | --- | --- | --- | --- |
| 17.00-27.00 | 544 | 36.50(34.80 ~37.20) | 35.60(34.00 ~37.20) | 36.50(34.50 ~37.30) | 36.50(35.20 ~37.20) | 14.02-19.07 | 531 | 36.50(35.20 ~37.20) | 36.70(34.70 ~37.30) | 36.70(35.20 ~37.20) | 36.50(35.20 ~37.20) |
| 27.00-30.00 | 760 | 36.70(35.00 ~37.20) | 36.20(34.20 ~37.20) | 36.50(35.00 ~37.20) | 36.70(35.30 ~37.20) | 19.07-20.43 | 536 | 36.70(35.00 ~37.20) | 36.50(34.70 ~37.20) | 36.50(34.80 ~37.20) | 36.70(35.30 ~37.30) |
| 30.00-31.00 | 320 | 36.50(34.80 ~37.20) | 36.00(34.20 ~37.20) | 36.00(34.20 ~37.00) | 36.80(35.30 ~37.30) | 20.43-21.64 | 523 | 36.70(35.00 ~37.20) | 36.20(34.50 ~37.20) | 36.50(34.80 ~37.20) | 36.80(35.30 ~37.30) |
| 31.00-34.00 | 622 | 36.80(35.30 ~37.20) | 36.50(35.00 ~37.20) | 36.80(35.50 ~37.20) | 36.80(35.30 ~37.30) | 21.64-23.51 | 527 | 36.70(35.20 ~37.20) | 36.50(34.20 ~37.20) | 36.50(35.20 ~37.00) | 36.80(35.50 ~37.30) |
| 34.00-50.00 | 398 | 36.50(34.80 ~37.20) | 36.60(34.70 ~37.30) | 36.50(35.00 ~37.20) | 36.70(35.00 ~37.20) | 23.51-39.00 | 527 | 36.50(34.80 ~37.20) | 36.00(34.50 ~37.20) | 36.50(35.00 ~37.20) | 36.70(35.00 ~37.20) |

**Abbreviation:** LG pattern, Low glucose pattern; MG pattern, Medium; HG pattern, High glucose pattern; PBMI, Pre-pregnancy Body Mass Index

**Supplementary Table 2.** The Relationship between glucose patterns and gestational weeks at delivery under different maternal characteristics.

| **Characteristics** | **β** | **95%CI** | ***P* for trend** |
| --- | --- | --- | --- |
| Age |  |  |  |
| Overall | 0.057 | (-0.009 ~0.124) | 0.091 |
| LG pattern | 0.029 | (-0.053 ~0.111) | 0.483 |
| MG pattern | 0.108 | (-0.037 ~0.252) | 0.145 |
| HG pattern | 0.224 | (0.036 ~0.413) | 0.020* |
| PBMI |  |  |  |
| Overall | -0.105 | (-0.170 ~-0.040) | 0.002* |
| LG pattern | -0.055 | (-0.134 ~0.023) | 0.169 |
| MG pattern | -0.059 | (-0.199 ~0.081) | 0.407 |
| HG pattern | -0.297 | (-0.487 ~-0.107) | 0.002* |

**Abbreviation:** LG pattern, Low glucose pattern; MG pattern, Medium; HG pattern, High glucose pattern; PBMI, Pre-pregnancy Body Mass Index

**p* for trend < 0.05

**
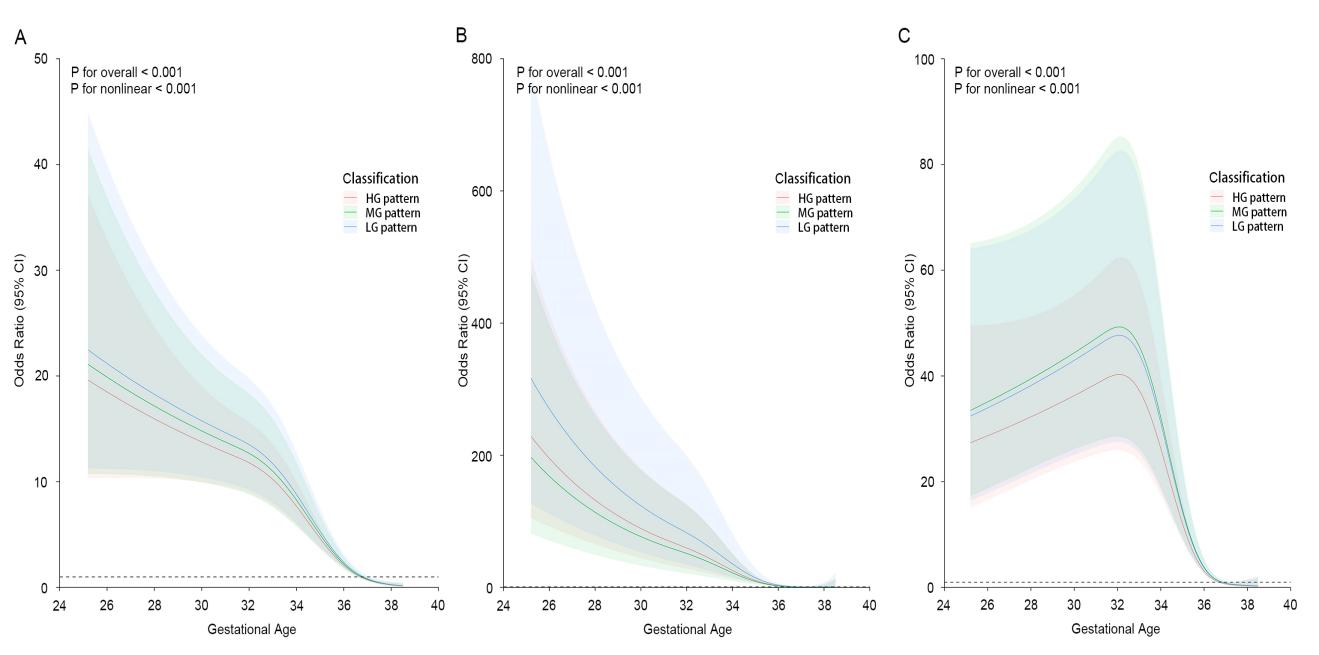
**

**Supplementary Figure 1.** The association between the gestational week of delivery in twin pregnancies under different blood glucose patterns and the ORs for various neonatal outcomes was determined by restricted cubic spline fitting. (A) For NICU admission. (B) For neonatal respiratory failure. (C) For neonatal hyperbilirubinemia. The OR reference level was the median week of gestation at delivery. The reference line is based on the median gestational week, and y-axis is set to 1. These curves have been adjusted for maternal age, PBMI, nulliparity, and primigravida status.
